# Development of environmental loop-mediated isothermal amplification (eLAMP) diagnostic tool for *Bulinus truncatus* field detection

**DOI:** 10.1186/s13071-023-05705-4

**Published:** 2023-02-28

**Authors:** Manon Blin, Bruno Senghor, Jérôme Boissier, Stephen Mulero, Olivier Rey, Julien Portela

**Affiliations:** 1 Hosts Pathogens Environment Interactions, UMR 5244, CNRS, IFREMER, UM, University of Perpignan, Via Domitia, 66860 Perpignan, France; 2 SAS ParaDev®, 66860 Perpignan, France; 3 VITROME, IRD-UCAD International Campus, 1386 Dakar, Senegal; 4 Univ. Grenoble-Alpes, Univ. Savoie Mont Blanc, CNRS-LECA, 38000 Grenoble, France

**Keywords:** Environmental DNA, LAMP assay, Intermediate host control, *Bulinus truncatus*

## Abstract

**Background:**

Global changes are reshaping the distribution of vector-borne diseases by spreading vectors to previously non-endemic areas. Since 2013, urogenital schistosomiasis has emerged in Corsica and threatens European countries. Gastropod vectors release schistosome larvae that can infect humans who come into contact with freshwater bodies. Monitoring schistosomiasis host vectors is a prerequisite to understand and subsequently to control this pathogen transmission. Because malacological surveys are time consuming and require special expertise, the use of a simple molecular method is desirable.

**Methods:**

The aim of this study is to develop a ready-to-use protocol using the LAMP (loop-mediated isothermal amplification) method to detect environmental DNA of *Bulinus truncatus*, vector of *Schistosoma haematobium*. Interestingly, LAMP method possesses all the characteristics required for adaptability to field conditions particularly in low-income countries: speed, simplicity, lyophilized reagents, low cost and robustness against DNA amplification inhibitors. We have tested this new method on Corsican water samples previously analysed by qPCR and ddPCR.

**Results:**

We demonstrate that our diagnostic tool *B. truncatus* eLAMP (Bt-eLAMP) can detect the eDNA of *Bulinus truncatus* as effectively as the two other methods. Bt-eLAMP can even detect 1/4 of positive samples not detectable by qPCR. Moreover, the complete Bt-eLAMP protocol (sampling, sample pre-process, amplification and revelation) does not require sophisticated equipment and can be done in 1 ½ h.

**Conclusions:**

LAMP detection of environmental DNA provides large-scale sensitive surveillance of urogenital schistosomiasis possible by identifying potentially threatened areas. More generally, eLAMP method has great potential in vector-borne diseases and ecology.

**Graphical Abstract:**

**Figure:**
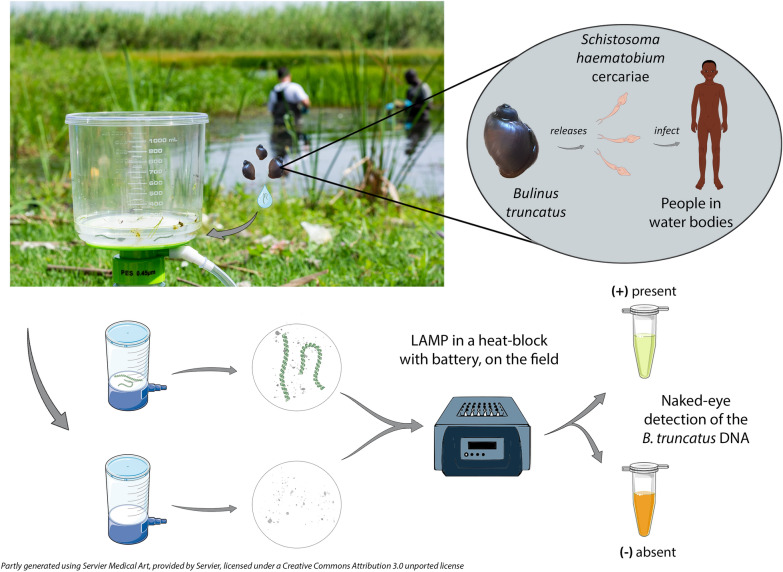

**Supplementary Information:**

The online version contains supplementary material available at 10.1186/s13071-023-05705-4.

## Background

It is well established that global changes increase the risk of infectious disease outbreaks [1–3]. The successive epidemic waves over the past decade such as the swine flu pandemic, numerous Ebola virus outbreaks in West Africa, 2015 Zika virus outbreak and the COVID-19 pandemic all resulted in significant mortality and morbidity while spreading to multiple countries [2]. The incidences of vector-borne diseases in humans and animals are impacted by climate change and human lifestyle (e.g. migration, pollution, urbanization) [1, 4, 5]. For several years, many of them have been emerging or re-emerging worldwide. For example, the geographical range of tick-borne encephalitis virus and the human incidence are increasing throughout Europe, including France, in areas that were previously non-endemic [6]. Although schistosomiasis is a disease linked to poverty and lack of potable water in households, schistosome infection has been identified in southern Europe (France and Spain) [7, 8]. Gastropod vectors release schistosome larvae that can infect humans who come into contact with freshwater bodies. In summer 2013, > 106 cases were diagnosed in Corsica, a French Mediterranean island known for its touristic attractiveness [9, 10]. Since then, a few cases of autochthonous transmission have persisted year after year [11]. For many parasitic diseases, global changes lead to a spatial redistribution of the vectors or the intermediate hosts which transmit the diseases and may also lead to disease emergence [12–16]. Mapping infectious disease outbreak areas is essential to both identify populations at risk of infection and communicate and warn the public [17]. Concerning vector-borne diseases, since the transmission is dependent on the presence of the vectors, mapping their spatial distributions is necessary for disease control. It has been shown in several African countries that snail control campaigns play a major role in controlling snail-borne diseases (SBDs) and that regular application of molluscicides probably contributes to the elimination of the SBDs in at-risk areas [18–20]. However, the monitoring of SBDs, particularly those transmitted via freshwater snail species, is less obvious. The spatial and temporal heterogeneity of the distribution of freshwater molluscs explains the difficulty of establishing these risk maps [21, 22]. Conventional sampling methods are therefore laborious and time-consuming, and they require malacologists for taxonomic identification, which have become increasingly scarce in recent years as molecular biology has taken off. Therefore, it is currently very difficult to monitor snail populations on a large spatial and temporal scale [21, 22].

The development of tools to simplify the monitoring of SBDs has been considered to address this issue, based on environmental DNA (eDNA) [23]. eDNA amplification methods were developed to detect many parasite vectors such as *Aedes albopictus* [24] and also snail hosts such as *Galba truncatula* and *Austropeplea tomentosa* [25, 26], both species being involved in the transmission of the liver fluke (*Fasciola hepatica*). The detection of eDNA is usually done using quantitative PCR DNA amplification technique. More recently, the eDNA detection of *Oncomelania hupensis* and *Bulinus truncatus*, involved in the transmission of *Schistosoma japonicum* and *Schistosoma haematobium*, respectively, has been made possible by either quantitative PCR (qPCR) or digital droplet PCR (ddPCR) [21, 27]. The results obtained in these last studies showed high-sensitivity and -specificity detection. Detection of *B. truncatus* eDNA by ddPCR allowed finding up to 0.06 copies/µl [21]. This represents a significant advance in the development of tools to simplify SBD monitoring. However, all these techniques require sophisticated and expensive equipment for both sample preparation (i.e. the use of expensive and time-consuming DNA extraction kits to obtain eDNA without PCR inhibitors) and amplification equipment (i.e. LightCycler or ddPCR). These costly techniques require a well equipped laboratory, which is rarely available in schistosome-endemic areas. Development of cost-effective and field-adapted tools for realization of snail distribution maps is still needed [17, 23, 28]. The loop-mediated isothermal amplification (LAMP) technique has the potential to make this possible [29–31]. Interestingly, the LAMP method possesses all the characteristics required for adaptability to field conditions particularly in low-income countries: speed, simplicity, lyophilized reagents, low cost and robustness against DNA amplification inhibitors [29, 30, 32]. Indeed, using a *Bst* polymerase enzyme with strand displacement activity and four to six primers hybridizing on six to eight regions of the target sequence, the exponential DNA amplification reaction can be performed at constant temperature, without prior denaturation of the DNA double strands. Indeed, the hybridization of the primers will create stem-loop structures on the newly amplified strands leading to the exponential multiplication of the number of new amplification start zones. We finally obtain a smear composed of several fragments of varied sizes which form a ladder after revelation by electrophoresis gel. Thus, the reaction is performed very quickly (usually < 1 h) at a constant temperature of around 63 °C in a simple heating block [29, 31, 33]. Since no large or expensive equipment is required to perform a LAMP, recent studies have focused on the development of portable devices to perform field diagnosis as close as possible to the sampling site [34–37]. Due to its robustness against inhibitors, it can be performed on samples with very simplified preparation, without the need for DNA extraction kits to purify the DNA, which can also be done in the field [38, 39]. DNA amplification can be detected by naked-eye visualization, gel electrophoresis or real-time fluorescence, offering flexibility of use in field settings [34, 36]. Recent studies have shown that it is possible to detect eDNA by LAMP [40–43]. An environmental LAMP (eLAMP) was developed to detect the presence of faecal indicator bacteria in water in 1 h, without sophisticated laboratory equipment or highly trained personnel [40]. Two teams succeeded in amplifying mollusc eDNA (*Galba truncatula* and *Dreissena* sp.) from water samples with LAMP [41, 42].

The aim of this study is to develop a rapid diagnostic LAMP tool to detect the environmental DNA of *Bulinus truncatus*, intermediate host of *Schistosoma haematobium*. Schistosomiasis is the second most common parasitic disease after malaria and remains a neglected tropical disease. Despite its high incidence (230 million infected people and 200,000 deaths per year [44]), efforts to conduct snail control campaigns are very low [28, 45, 46]. However, in 2017, WHO called for refocusing on snail control to sustain progress, calling on member states to develop or adapt national vector control strategies, aiming to reduce the incidence of vector-borne diseases, including schistosomiasis, by at least 40% by 2025 [46]. Thus, cost-effective, field-appropriate tools for rapid mapping of snail distribution are needed for this disease [23]. Compared to qPCR or ddPCR amplification methods, the eLAMP seems advantageous for field applicability in low-income countries. However, this advantage disappears if the DNA extraction protocol that precedes amplification is not adapted to field conditions like using DNA extraction kits. Our work consisted in (i) development of *B. truncatus*-specific eLAMP assay, (ii) comparison of the results obtained by LAMP on an eDNA bank with those obtained by qPCR and ddPCR and (iii) development of a full field-friendly protocol of detection.

## Methods

### Material

The *Bulinus truncatus* eLAMP (Bt-eLAMP) diagnostic tool was developed from both an environmental DNA (eDNA) bank and newly extracted DNA from molluscs.

The eDNA bank is composed of 200 samples previously collected on Corsica island, France [21]. These samples were DNA extracted from water filtration membranes collected in two distinct rivers from southern Corsica. Urogenital schistosomiasis transmission has recently been evidenced in these two rivers [9, 10, 47]. Three different volumes of water (1, 3 and 5 l) from both the shore and the streambeds of the rivers were collected. Four sites were sampled in the Cavu River: the Mulinu bridge and Tyroliana park sites (sampled in duplicate), the three pools site where *B. truncatus* are present and the water intake site where *B. truncatus* are absent. A single site was sampled from the Solenzara River where *B. truncatus* are present. Negative controls were made by filtering pure spring water along the river. In addition to these sampling sites in Corsica, eDNA was also sampled from an artificial channel within the campus of Perpignan University, where *B. truncatus* has never been recorded, as a negative control. The eDNA extraction protocol from the resulting water filtration membranes was previously described [21] and is briefly recalled here: each filtration membrane was divided into four quarters and extracted using DNeasy^®^ Blood & Tissue Kit (QIAGEN). From the 200 samples, 144 eDNA samples were collected where *B. truncatus* were present and 56 eDNA samples collected where *B. truncatus* were not present, and they were amplified using qPCR. A sample was considered positive if at least one quarter membrane was positive. The ddPCR reactions were run on pools of DNA extracts from the four quarters of each membrane (i.e. 50 reactions) [21]. Results per membrane obtained with both qPCR and ddPCR were compared to eLAMP (present study). Results per membrane quarter obtained by qPCR were also compared to eLAMP.

In addition to eDNAs, and to test the specificity of our primer sets, DNA from freshwater snails (*Bulinus* sp. and other phylogenetically related genera) was also extracted using the DNeasy^®^ Blood & Tissue Kit (QIAGEN) following the tissue extraction protocol. Briefly, after grinding each snail, the samples were put in a Speedvac™ DNA 130 (Savant™, Thermo Scientific™) at 65 °C for 20 min to remove any alcohol or water residuals. The snails were then lysed at 56 °C for 4 h in a solution containing 20 ml proteinase K and 180 ml ATL buffer. The remaining steps were performed according to the manufacturer's instructions. The DNA concentration was measured using a Qubit^®^ 2.0 fluorometer. These last DNAs were diluted with ultrapure water to obtain final concentrations of 0.5 ng/µl. The resulting DNA extracts and dilutions were then stored at -20 °C until used.

### eLAMP primer set design

The DNA target selection was exhaustive to maximize the chance of success in the development of the eLAMP diagnostic tool. The ITS2; 5,8S; 18S; COI; and 28S *B. truncatus* DNA regions were downloaded from GenBank database (NCBI, https://www.ncbi.nlm.nih.gov/genbank/; accession numbers: MH361757 for 5,8S; KJ157340 for 18S [48]; KJ157370 for 28S [48]; MT707426 for COI [49] and MG757890 for ITS2 [50]). When available, sequences from other *Bulinus* species were downloaded from GenBank database to develop a *B. truncatus*-specific detection tool. The design of primer sets was carried out using Primer Explorer V5 software (Eiken Chemical Co., Ltd., Japan; http://primerexplorer.jp/e/) following the criteria described in "A Guide to LAMP primer designing" (https://primerexplorer.jp/e/v5_manual/index.html). The sets had to meet the following parameters: length of each primer between 18 and 22 bp; Tm between 64 and 67 °C for F1c/B1c pair; Tm between 59 and 62 °C for F2/B2 and F3/B3 pairs; GC rate between 50 and 65%; dG threshold < –4 kcal/mol for 5’ and 3’ stability and between 0 and –1 kcal/mol for dimer check. Primer dimer estimation and stability of each primer set were checked online using the "Multiple Primer Analyzer" (Thermo Scientific Web Tool) and OligoAnalyzer™ Tool (IDT™). All the primers selected were synthesized by Eurogentec (Seraing, Belgium).

### LAMP reaction

For each designed primer set several amplification assays were performed by adjusting the amplification reaction parameters (i.e. MgSO_4_, primers and betaine concentration, reaction time and temperature). The selected protocol was as follows: a total volume of 10 µl containing 1.2 µM of the internal primers FIP and BIP, 0.2 μM of the external primers F3 and B3, 0.4 µM of the LOOP primers LB and LF, 1.0 mM of each dNTP (New England Biolabs), 1X Isothermal Amplification Buffer II reaction buffer [20 mM Tris–HCl, 10 mM (NH4)2SO4, 150 mM KCl, 2 mM MgSO_4_, 0. 1% Tween^®^ 20, pH 8.8@25 °C (New England Biolabs, UK)], 3 mM additional MgSO_4_ (New England Biolabs, UK), 1.2 M betaine and 1 U of *Bst* 2.0 WarmStart DNA polymerase (New England Biolabs, UK) along with 2.5 µl DNA template. Reaction tubes were placed in a battery-operated heating block at 63 °C for 45 min followed by an enzyme inactivation phase at 80 °C for 5 min if the results were not visualised immediately after the reaction. Result visualisations were done according to different protocols classically used in LAMP DNA amplification: (i) final point visual detection of fluorescence after adding 2 μl of 1:50 diluted 10,000× concentration of SYBR Green (Invitrogen) (green: positive; orange: negative); (ii) final point visual detection of the LAMP products on 2% agarose gel electrophoresis with 2 µl MIDORI Green Advance (Nippon Genetics) for 50 ml agarose gel, revealed by UVs; (iii) real-time detection by incorporating Sybr-Green 0.25× in the reaction mix and monitoring the amplification in a Genie III portable device (Optigene Ltd).

### Specificity and limit of detection

The specificity of all designed primer sets was assessed on DNA templates from 12 freshwater snail species (Table 1), including four specimens of the same genus but different species (i.e. *B. forskalii*, *B. globosus*, *B. umbilicatus* and *B. senegalensis*) and other phylogenetically related genera. *Bulinus truncatus* from five different localities (Table 1) were evaluated to visualise a possible intraspecific variability. Only the primer set amplifying exclusively *B. truncatus* DNA was kept for the ensuing experiments.

**Table 1:** Snail species used for assessing the specificity of the developed *Bulinus truncatus* LAMP primer sets

Snail species	Origin	Sampling date
*Bulinus truncatus*	Diama, Senegal	2022
	Lampsar, Senegal	2022
	Mbane, Senegal	2022
	Corsica, France	2018
	Saneinte, Senegal	2022
*Bulinus globosus*	Lampsar, Senegal	2022
*Bulinus senegalensis*	Podor, Senegal	2022
*Bulinus forskalli*	Khodit, Senegal	2022
*Bulinus umbilicatus*	Barkedji, Senegal	2022
*Biomphalaria glabrata*	Brazil	2018
*Bithynia tentaculata*	Corsica, France	2018
*Ancylus fluviatilis*	Corsica, France	2018
*Gyraulus laevis*	Corsica, France	2018
*Physa acuta*	Corsica, France	2018
*Potamopyrgus antipodarum*	Corsica, France	2018
*Pisidium casertanum*	Corsica, France	2018

The limit of detection (LOD) was assessed by LAMP assays on DNA from *B. truncatus* tenfold serially diluted from 10 ng/µl to 1.10^–9^ ng/µl before and after adding LOOP primers to the reaction. From the detection limit DNA concentration obtained, the number of *B. truncatus* target copies per microlitre was estimated using the following equation:

$${N}_{c}=\frac{\mathrm{C}\times \mathrm{V}\times {1.10}^{-9}}{\mathrm{M}\times {S}_{g}\times 2}{ \times N}_{A}\times {N}_{rDNA}$$

where N_c_ = the number of *B. truncatus* target copies per microlitre; C = limit detection DNA concentration in ng/µl; V = volume (here, 1 µl); M = average molar mass of a nitrogenous base = 309 g/mol; Sg = *B. truncatus* genome size = 1.2.10^9^ pb; N_A_ = Avogadro number; N_rDNA_ = average rDNA copy number in a genome = 93 copies [51–56].

### Field applicability

We have developed a new field-friendly eDNA concentration protocol allowing us to take full advantage of the field benefits of LAMP. The water is collected in a clean plastic commercial bottle on the surface of the water body. Then, 500 ml to 1500 ml of water is filtered along the river using a 0.45 µm filtration unit (PES VWR 1L 514-0301) and a manual pump. The water is filtered until the membrane is blocked because of sediment stacking. The membrane is cut in four quarters with a scalpel blade and deposited in a single 25-ml Falcon™ tube containing 15 ml lysis buffer [100 mM NaCl, 250 mM EDTA, 5% SDS and 10 mM Tris–HCl (pH = 8)]. Clamps used to remove the membranes from filtration units are decontaminated before each sampling by successively placing them in a 10% bleach bath for 15 s and then 15 s in a DNA AWAY™ solution (Thermo Scientific). After vigorous agitation, the liquid is collected with a 20-ml syringe. The liquid is pre-filtered with a 40-µm membrane to remove the larger sediments and then filtered with a 0.45-µm (13-mm) Whatman^®^ GD/XP syringe filter (PTFE Sigma Aldrich WHA69742504). The last filter is washed with 5 ml distilled water and dried with the same volume of air. The eDNA is finally recovered by reverse filtration with 500 µl ultrapure water thanks to a syringe coupled with a conical 18 GA dispensing needle (Metcal).

This last protocol was assessed in two Senegalese water bodies investigated in July 2022. The Lampsar site (16°06′29′′N; 16°20′59′′W) and Diama site (16°12′36′′N; 16°24′10′′W) are both situated along the Senegal River in Saint-Louis region, Senegal. At each site, water samples were collected in triplicate with about 10 m between samplings. Seven hundred millilitres, two times 500 ml and three times 500 ml were collected for Lampsar and Diama sites, respectively. At each site negative controls were carried out by filtering 1500 ml pure spring water strictly following the same procedure as for biological samples. The presence on site of *B. truncatus* was verified by sampling molluscs for 1 h by two malacologists.

### Statistical analysis

The agreement between results obtained with the newly developed LAMP and the qPCR for the detection of *B. truncatus* DNA on the 200 field eDNA library were statistically analysed calculating a kappa coefficient analysis using psych package (version 2.2.9) in R software. Field sensitivities on the eDNA bank were calculated with a 95% confidence interval using stats package (version 3.6.2) in R software.

## Results

### Specificity and limit of detection of selected LAMP primer sets

A total of 18 primer sets were designed. After parameter adjustment and sensitivity and specificity assessment, we have selected the best candidate which targets the ITS2” marker (Table 2). An additional file shows the primer alignment on the ITS2 sequence (see Additional file 1). Primer dimer minimum dG for this set was –0.93 kcal/mol.

**Table 2:** Primer sequences designed for *Bulinus truncatus* eLAMP assays

Target	Primers	Sequences (5’-3’)	Sequence length (bp)	Melting temperature (°C)
Internal transcribed spacer 2 (ITS2)	F3	GTCCTTCCTGTCCCCCTT	18	59.79
	B3	CGTCACATGTCCACCTCACA	20	61.59
	F2	ACGCATGTCGCCGTTAC	17	59.33
	F1c	AAGAGCACGAGCCGGGTCC	19	66.28
	B2	CGCGTTATAGCCCATCTCAA	20	59.37
	B1c	GCGAGTCATACGGACCTCGTG	21	64.21
	LB	CACAGTCTCTCCCCCACACC	20	63.67
	LF	CCTGGCGACGAGAGATCACC	20	63.89

**Fig. 1:**
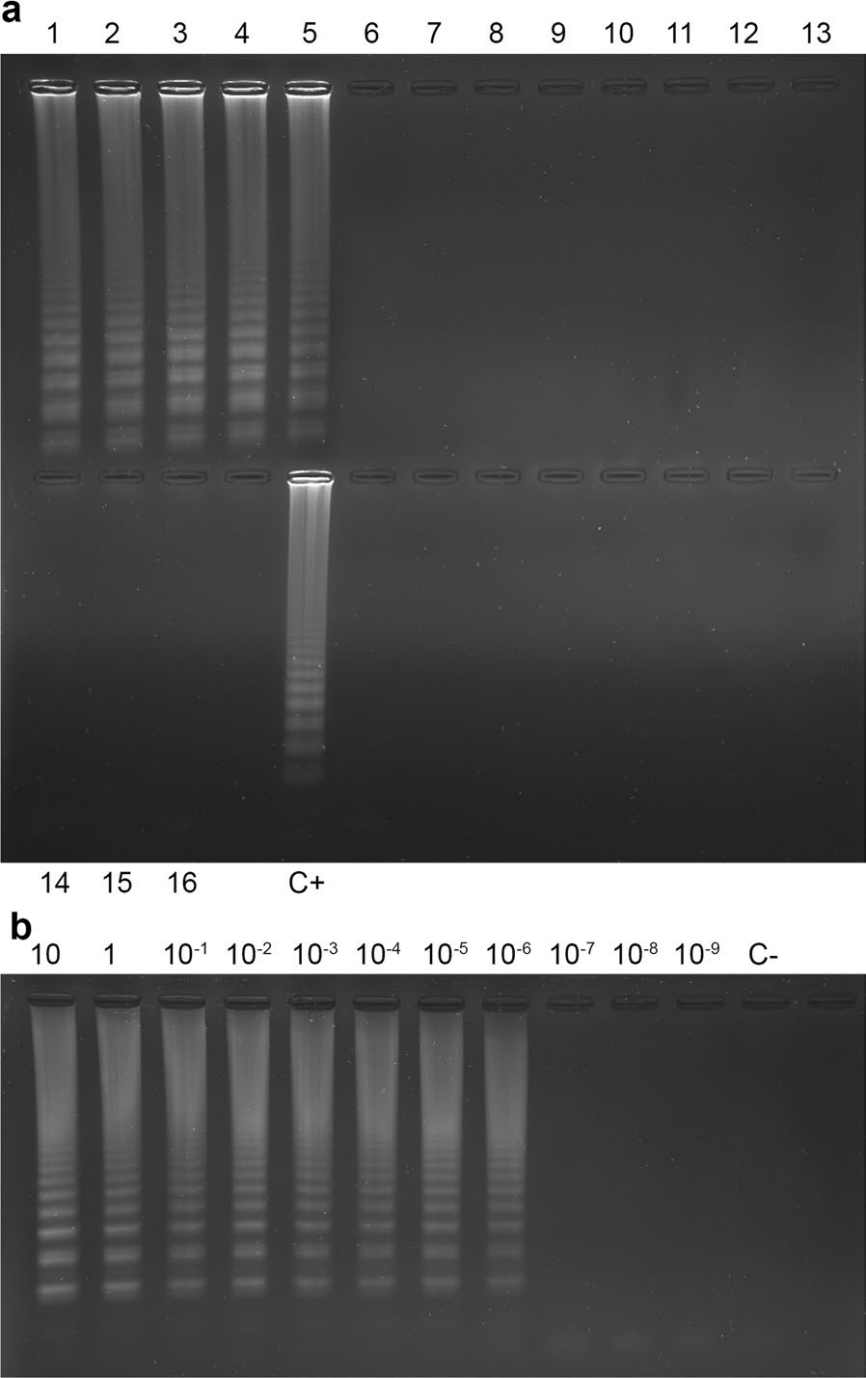
Specificity and LOD (limit of detection) of the *Bulinus truncatus* eLAMP (Bt-eLAMP) assay. Results obtained with agarose gel electrophoresis after 45 min of reaction. **a** Specificity; lanes 1–5, *Bulinus truncatus* DNA templates from five different localities (Diama, Lampsar, Mbane, Corsica and Saneinte); lane 6, *Bulinus globosus*; lane 7, *Bulinus senegalensis*; lane 8, *Bulinus forskalli*; lane 9, *Bulinus umbilicatus*; lane 10, *Biomphalaria glabrata*; lane 11, *Bithynia tentaculata*; lane 12, *Ancylus fluviatilis*; lane 13, *Gyraulus laevis*; lane 14, *Physa acuta*; lane 15, *Potamopyrgus antipodarum*; lane 16, *Pisidium casertanum*; lane C + , positive control (*Bulinus truncatus* DNA; 1 ng); lane C-, negative control (water as template). **b** LOD; lane 1–11, *Bulinus truncatus* DNA tenfold serial dilutions (10 ng/μL to 1 ag/μl); lane C-, negative control (water as template)

Bt-eLAMP specificity was assessed by testing extracted DNA from 11 non-targeted species and five targeted molluscs from different localities. Figure 1a shows that only the five *B. truncatus* DNA samples are amplified. Sensitivity test (Fig. 1b) shows that the LOD is 1 fg/µl (i.e. equivalent to about Nc = 0.07 copies per microlitre) with complete primer set (including LOOP primers). The detection limit was 1 pg/µl before adding the LOOP primers.

### Comparison of LAMP to qPCR and ddPCR amplification methods on eDNA samples

**Fig. 2:**
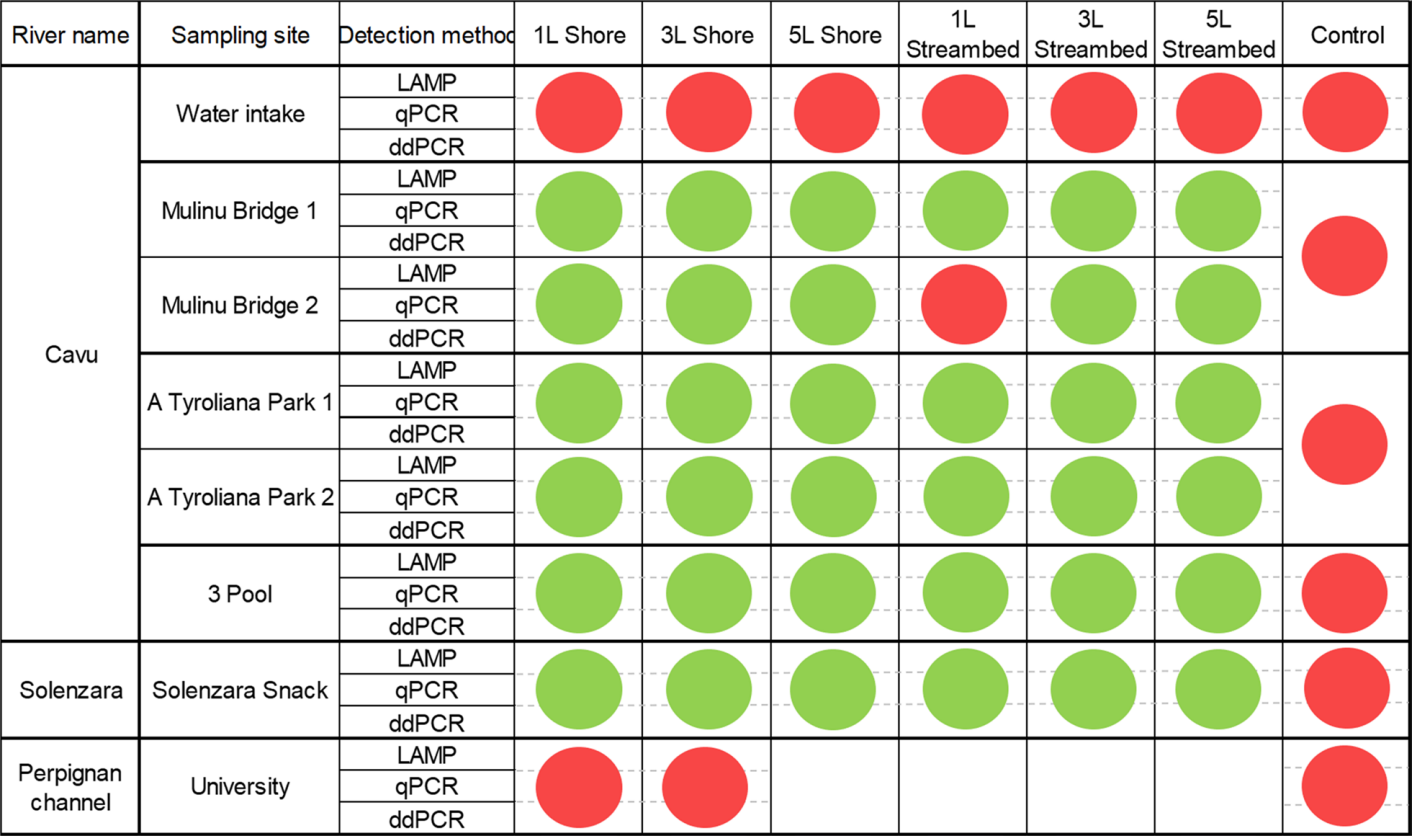
Comparison of *Bulinus truncatus* environmental DNA detection by loop-mediated isothermal amplification (LAMP), quantitative polymerase chain reaction (qPCR) and droplet digital polymerase chain reaction (ddPCR) amplification methods. Each cell represents DNA amplification results from eDNA collected in two Corsican rivers (Cavu and Solenzara) and Perpignan Channel; *Bulinus truncatus* snails are present in all sites except the water intake site in the Cavu River and university site in the Perpignan Channel. Three volumes of water (1, 3 and 5 l) from both the shore and the streambeds of the rivers were collected in all Corsican sites. Each line represents the amplification result from each site using LAMP, qPCR or ddPCR amplification method. qPCR and ddPCR results come from Mulero et al. [21]. A green membrane means that at least one quarter of the filtration membrane is positive for *Bulinus truncatus* presence and red membrane means that no quarter of the filtration membrane is positive. Empty cells mean that no filtration was done under these conditions

Figure 2 shows detection accuracy comparison among LAMP, qPCR and ddPCR amplification methods on eDNA samples collected from two Corsican rivers. The Bt-eLAMP method provided the same results as those obtained using the qPCR and ddPCR. All membranes are positive except for the "1LStreambed" membrane from the Mulinu bridge two site. No positive signal was detected from the six field negative controls or from the University site where *B. truncatus* never occurred or for samples collected at Water intake site from the Cavu River where no *B. truncatus* were collected (Fig. 2). Considering results obtained per membrane quarter where *B. truncatus* was present, 110 of 144 samples were successfully amplified with LAMP; meanwhile, 91 of 144 samples were successfully amplified with qPCR. The sensitivities of LAMP and qPCR methods were estimated to be 76.4% (95% CI 68.6–83.1) and 63.2% (95% CI 54.8–71.1), respectively. Kappa agreement obtained with these results is equal to 0.51 (Table 3).

**Table 3:** Cross table comparing LAMP and qPCR analyses of *Bulinus truncatus* eDNA with kappa agreement

No. of samples				
qPCR results	LAMP positive	LAMP negative	Total	Kappa value
Positive	76	15	91	0.51
Negative	34	75	109	
Total	110	90	200	

### Field applicability

**Fig. 3:**
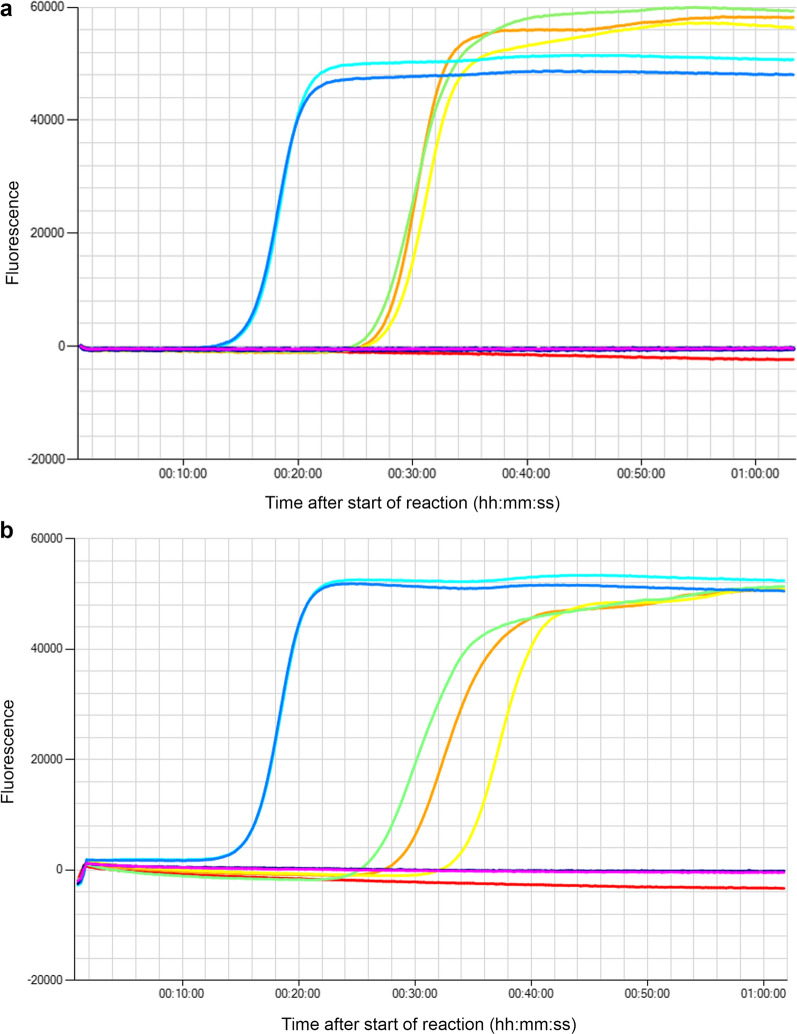
Real-time loop-mediated isothermal amplification of *Bulinus truncatus* environmental DNA in Diama (a) and Lampsar (b) sites, Senegal. Orange, yellow and green lines are tested samples. Turquoise and blue lines are positive controls (1 ng of *B. truncatus* extracted DNA). Red line is field negative sample (1500 ml filtration of mineral water). Violet and pink lines are laboratory negative samples (ultrapure water)

The use of LAMP amplification as field-friendly detection method was improved by developing a new eDNA concentration protocol that could be done along the water bodies. The entire protocol was tested on Lampsar and Diama sites, Senegal. In these two sites, respectively, 95 and 229 *B. truncatus* were sampled by two malacologists for 1 h sampling. Four of the 95 *B. truncatus* sampled from Lampsar site emitted cercariae after 2 h light exposure. *Bulinus globosus* was present at both sites. *Bulinus forskalii* was only present at Diama site, while we found *Biomphalaria pfeifferi* only at Lampsar site. The three eDNA samplings from the two sites were positive (Fig. 3) showing 100% accuracy of the method. None of the negative controls were positive. Samples of *B. truncatus* eDNA were amplified on average 12 min after amplification of the positive controls (*B. truncatus* extracted DNA).

## Discussion

Global changes are reshaping the distribution of vector-borne diseases by spreading vectors to previously non-endemic areas. Therefore, the development of cost-effective and field-adapted tools for vector distribution maps is necessary [17, 28]. In the present study, we have developed a rapid LAMP diagnostic tool to detect the environmental DNA of a freshwater snail vector (i.e. *B. truncatus*) of a tropical parasite (i.e. *S. haematobium*). Our Bt-eLAMP successfully amplified an ITS2 DNA marker fraction of *B. truncatus* after 45 min of incubation at 63 °C. In vitro assays evidenced the high sensitivity of the Bt-eLAMP with a LOD equal to 1 fg/µl when it included LOOP primers. Considering that the LOD was 1 pg/µl before adding the LOOP primers, we deduce that the addition of LOOP primers allows a gain in sensitivity of a factor of 1000. From a methodological point of view, this highlights the importance of designing primer sets allowing inclusion of LOOP primers. The detection limits of the developed LAMP techniques are generally between 100–0.1 fg/µl [43, 57–59]. Our LOD is lower compared to the LOD previously evaluated for the LAMP eDNA detection of the freshwater snail vector of fasciolosis (*Galba truncatula*) [41]. These last authors measured a 0.349 pg/μl LOD [41]. When estimating the copy number equivalence (0.07 copies per microlitre) with the complete primer set (i.e. including LOOP primers), we found a sensitivity close to that obtained using ddPCR (0.06 copies per microlitre) [21]. Our Bt-eLAMP showed equal sensitivity compared to the ddPCR, which is considered the most sensitive DNA amplification technique of all [60, 61].

Our Bt-eLAMP assay showed 100% specificity for *B. truncatus* even considering closely related snail species. This suggests that the Bt-eLAMP assay is robust enough to detect *B. truncatus* eDNA in areas where other snails co-occur without risk of cross-reactivity. Our tool can also allow species determination between *B. truncatus* and other *Bulinus* species such as *B. globosus*, which are morphologically indistinguishable. On the other hand, the usefulness of this high specificity could be questioned if we consider that some molluscs’ sister species, such as *B. globosus*, could also be involved in the transmission of urogenital schistosomiasis [62]. It would be interesting to also develop eDNA diagnostic tools allowing a pan-specific detection of both *B. truncatus* and *B. globosus* or all species of the *Bulinus* genus. The difficulty will be to find an equilibrium between sufficiently conserved regions between *Bulinus* species and sufficiently different from other gastropods to obtain a genus-specific primer set. This last task is more difficult as the number of primers required in LAMP and the positioning constraints between them on the target sequence increase the length of the sequence to be found meeting these requirements.

Considering our eDNA bank, the Bt-eLAMP method provided exactly the same overall detection capacity compared to the qPCR or the ddPCR [21]. Considering each membrane quarter, with 76.4% (95% CI 68.6–83.1) and 63.2% (95% CI 54.8–71.1) of positive detection using LAMP or qPCR, respectively, the LAMP method showed higher detection capacity compared to qPCR [21]. The kappa coefficient highlights moderate agreement between the two methods. Fifteen membrane quarters are negative in LAMP while they are positive in qPCR; conversely, 34 membrane quarters are negative in qPCR and positive in LAMP. The presence of PCR inhibitors could explain these last results. Indeed, it has already been proven that, in contrast to qPCR, LAMP is robust against inhibitors [29, 63].

Development of field friendly amplification methods such as LAMP need to be accompanied by the development of a field-friendly pre-process of the sample. Use of DNA extraction kits that have a low yield due to their high purification level requires special apparatuses such as centrifuges, which are costly. Therefore, we have developed a new field-friendly water sample pre-process to concentrate the eDNA. The sampling and pre-process protocol were done in 45 min in the field without electricity, which represents a significant advance in SBDs monitoring. Nevertheless, several improvements can be made to our protocol, especially on the water filtration step. The manual pump used limits the speed and convenience of the protocol since it took 30 min per sample to filter just 500 ml of water with difficulties. A 12-V battery-powered diaphragm water pump coupled with an adapted filter capsule was used to filter up to 50 l water in a DNA survey targeting trematodes in water [64]. To further enhance the practicability and speed of the Bt-eLAMP assay, an improved battery-operated pump could replace the manual pump. The compatibility between a motorised pump and the filter units used in our protocol needs to be tested.

We used the Genie III to obtain our field results from Senegal, which is a useful, easily transportable, battery-operated field tool, allowing visualization of reaction kinetics. Coupled with freeze-dried reagents, this type of portable device makes it possible to carry out the entire protocol (sampling, pre-process, amplification and revelation) in the field [32]. We should use another portable device for low-income countries where the use of the Genie III may be a financial obstacle. The COVID-19 global pandemic involved the development of several tools to perform low-cost and field-friendly LAMPs [34, 35, 65–67]. A low-cost and open-source LAMP diagnostic device has been developed that allows real-time amplification and fluorescence reading in the same way as the Genie III [34]. It has even been proven that the LAMP reaction can be done using a coffee capsule filled with phase change material, called a “T-cup”, allowing incubation at 63 °C in boiling water and a naked-eye revelation [35]. In another study, the revelation step of a smartphone-based LAMP could be assessed with a smartphone application coupled with filters allowing the fluorescence reading. In the future, the current LAMP test should be improved with freeze-dried reagents and this type of device and then tested on a large sampling.

## Conclusions

In this study, we developed a full field-friendly LAMP assay detecting the presence of the trematode intermediate snail host *B. truncatus* DNA in the environment. Due to LAMP's advantages of being field-friendly and allowing cost-effective pre-process samples, Bt-eLAMP test may be a valuable new tool for quickly and efficiently establishing recurrent risk maps for urogenital schistosomiasis. eLAMP has also been used to detect the presence of intestinal parasites in wastewater during treatment, even offering the possibility of looking for *Schistosoma sp.* eDNA directly [68]. It would be necessary to develop eLAMP for other *Bulinus* or *Biomphalaria* species responsible for the transmission of *Schistosoma*. eLAMP could also be developed for other SBDs that have the same diagnostic pressures as schistosomiasis.

## Supplementary Information

**Additional file 1.** Alignment of the Bt-eLAMP primers to the ITS2 marker (accession number: MG757890). Each arrow represents a primer. The green shades represent the direction of the primer hybridization (dark green: forward; light green: backward).

## Data Availability

All data generated or analysed during this study are included in this published article.

## Abbreviations

SBD: Snail-borne disease
LAMP: Loop-mediated isothermal amplification
eLAMP: Environmental loop-mediated isothermal amplification
PCR: Polymerase chain reaction
qPCR: Quantitative polymerase chain reaction
ddPCR: Droplet digital polymerase chain reaction
eDNA: Environmental DNA
LOD: Limit of detection
C+: Positive control
C−: Negative control
