# Supplementary material for: Development of environmental loop-mediated isothermal amplification (eLAMP) diagnostic tool for Bulinus truncatus field detection
Source: Parasit Vectors. 2023 Feb 28;16:78. doi: 10.1186/s13071-023-05705-4 (PMC9972309; doi:10.1186/s13071-023-05705-4)
Supplement: Supplementary file 1 — Additional file 1. Alignment of the Bt-eLAMP primers to the ITS2 marker (accession number: MG757890). Each arrow represents a primer. The green shades represent the direction of the primer hybridization (dark green: forward; light green: backward). [file 13071_2023_5705_MOESM1_ESM.docx]

Additional file 1


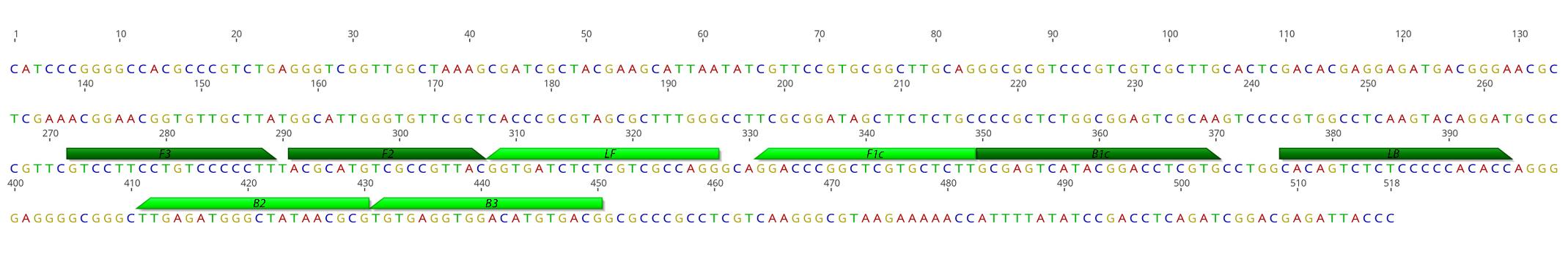


Additional file 1: Alignment of the Bt-eLAMP primers to the ITS2 marker (Accession number: MG757890). Each arrow represents a primer. The green shades represent the direction of the primer hybridization (dark green: forward; light green: backward).
